# Microbial Proteases Applications

**DOI:** 10.3389/fbioe.2019.00110

**Published:** 2019-06-12

**Authors:** Abdul Razzaq, Sadia Shamsi, Arfan Ali, Qurban Ali, Muhammad Sajjad, Arif Malik, Muhammad Ashraf

**Affiliations:** ^1^State Key Laboratory of Cotton Biology, Key Laboratory of Biological and Genetic Breeding of Cotton, The Ministry of Agriculture, Institute of Cotton Research, Chinese Academy of Agricultural Sciences, Anyang, China; ^2^School of Medicine, Medical Sciences and Nutrition, The Institute of Medical Sciences, University of Aberdeen, Aberdeen, United Kingdom; ^3^1-FB, Genetics, Four Brothers Group, Lahore, Pakistan; ^4^Institute of Molecular Biology and Biotechnology, The University of Lahore, Lahore, Pakistan

**Keywords:** microbial proteases, proteolytic enzymes, bacterial enzymes, industrial enzyme, substrate-specific proteases

## Abstract

The use of chemicals around the globe in different industries has increased tremendously, affecting the health of people. The modern world intends to replace these noxious chemicals with environmental friendly products for the betterment of life on the planet. Establishing enzymatic processes in spite of chemical processes has been a prime objective of scientists. Various enzymes, specifically microbial proteases, are the most essentially used in different corporate sectors, such as textile, detergent, leather, feed, waste, and others. Proteases with respect to physiological and commercial roles hold a pivotal position. As they are performing synthetic and degradative functions, proteases are found ubiquitously, such as in plants, animals, and microbes. Among different producers of proteases, *Bacillus* sp. are mostly commercially exploited microbes for proteases. Proteases are successfully considered as an alternative to chemicals and an eco-friendly indicator for nature or the surroundings. The evolutionary relationship among acidic, neutral, and alkaline proteases has been analyzed based on their protein sequences, but there remains a lack of information that regulates the diversity in their specificity. Researchers are looking for microbial proteases as they can tolerate harsh conditions, ways to prevent autoproteolytic activity, stability in optimum pH, and substrate specificity. The current review focuses on the comparison among different proteases and the current problems faced during production and application at the industrial level. Deciphering these issues would enable us to promote microbial proteases economically and commercially around the world.

## Introduction

Proteases are a universal entity that is found everywhere, namely, in plants, animals, and microbes. The peptide bond present in the polypeptide chain of amino acids is hydrolyzed by means of proteases (Barrett and McDonald, 1986). Proteases are degradative enzymes and show specificity and selectivity in protein modification (Rao et al., 1998). In the industrial sector, *Bacillus* sp. are the most active and dynamic extracellular alkaline protease producer. Of the three largest groups of industrial enzymes, proteases are one of them, and their global market is drastically increasing annually. Of the 60% of enzymes marketed worldwide, proteases account for 20% (Kang et al., 1995; Rao et al., 2009; Singhal et al., 2012). Proteases are an integral component of existing life on earth, such as animals, plants, and microbes. By a process of fermentation, proteases can be isolated and purified in a relatively shorter period of time, exhibiting high substrate specificity and catalytic activity (Kumar and Takagi, 1999; Rifaat et al., 2007; Singhal et al., 2012). It is estimated that proteases account for 1–5% of the genome of infectious organisms and 2% of the human genome (Puente et al., 2003). According to researchers, proteases control the activation, synthesis, and turnover of proteins to regulate physiological processes (Rawlings et al., 2004). Different physiological processes, such as formation, birth, aging, and even death are regulated by proteases (Chou et al., 1997, 2000, 2003; Chou and Howe, 2002; Chou, 2004, 2006). Proteases are vital in the imitation and spread of infectious diseases, and because of their significant role in the life cycle, they are imperative for drug discovery. In more than 50 human proteases, a single amino acid mutation may lead to a hereditary disease (Chou et al., 1998). Proteases are involved in normal and pathophysiological processes or conditions. This involvement of proteases may lead them to produce a therapeutic agent against deadly diseases, such as cancer and AIDS (Rawlings et al., 2004). Proteases similar in sequences and structures are grouped into clans and families, which are available in the MEROPS database (Kumar and Takagi, 1999). The proposed review highlights the proteolysis, function, and wide range of sources among different bacteria of microbial proteases. It also discusses the broad range of applications and upcoming advancement for the discovery of new and fresh proteases, especially alkaline proteases from bacteria (Reddy et al., 2008; Haddar et al., 2009a).

## Microbial Proteases

Proteases have been successfully produced by researchers from different microbial sources. Microbes account a two-thirds share of commercial protease around the globe (Beg and Gupta, 2003). Since the advent of enzymology, microbial proteolytic proteases have been the most widely studied enzyme. These enzymes have gained interest not only due to their vital role in metabolic activities but also due to their immense utilization in industries (Rao et al., 1998; Sandhya et al., 2005; Younes and Rinaudo, 2015). The proteases available in the market are of microbial origin because of their high yield, less time consumption, less space requirement, lofty genetic manipulation, and cost-effectiveness, which have made them suitable for biotechnological application in the market (Nisha and Divakaran, 2014; Ali et al., 2016). These microbial proteases are preferred to plant and animal proteases because of the presence of all desired characteristics for industrial applications (Palsaniya et al., 2012; Sathishkumar et al., 2015). Proteolytic enzymes found in microbes and mammalian systems are small in size, dense, and structurally spherical (Oberoi et al., 2001). Among different producers of alkaline proteases, Bacillus sp. is of immense importance (Rifaat et al., 2007). The proteases isolated from these microbial sources have a large number of dilutions in various industrial sectors (Pastor et al., 2001; Beg and Gupta, 2003; Das and Prasad, 2010). Usually, extracellular alkaline proteases are secreted out from the producer into the liquid broth from where these proteases are simplified and purified through down streaming to produce an end product. Comparatively, proteases produced by plants and animals are more labor-intensive than microbially produced proteases (Gupta et al., 2002; Kalaiarasi and Sunitha, 2009). Proteases produced by microbial sources are classified into groups based on their acidic or basic properties. They are also classified based on the presence of functional groups and the position of peptide bond (Gessesse, 1997; Panda et al., 2013). Microbial proteases are the most commercially exploited enzyme worldwide. A large number of intracellular proteases are produced by microbes playing a vital role in differentiation, protein turnover, hormone regulation, and cellular protein pool, whereas extracellular proteases are significant in protein hydrolysis (Rao et al., 1998; Johnvesly and Naik, 2001; Adrio and Demain, 2014), such as in processing of photographic film (Kumar and Takagi, 1999; Patil and Chaudhari, 2009), enzymatic synthesis on the basis of solvent and detergent preparation (Simkhada et al., 2010a), substrate specificity (Soroor et al., 2009), thermal tolerance (Amoozegar et al., 2007), and production of zein hydrolysates (Miyaji et al., 2006; Dodia et al., 2008; Jaouadi et al., 2008).

## Keratin

Keratins are proteins that are usually present in two forms, namely, hard keratins and soft keratins. Hard keratins mainly include the structural proteins that are prevalently present in fingernails, horns, beaks, upper layer of skin, and mainly hair. Fibers of the keratin proteins are self-assembled into compact follicles that make up the structure of hair. The process of assembling keratin proteins into a complex hair is under the control of multiple genes, cytokines, and growth factors (Charles et al., 2008). In contrast to hard keratins, soft keratins are those that are abundantly present in tissues, such as epithelial tissues. The structure of wool keratin shows great similarity to hair keratin. Three types of hair keratin have been known (Cheng et al., 1995). The first one is the alpha keratins; these range in size from 60 to 80 kDa. Having low sulfur content, these comprise mainly of alpha-helical domains. Overall, alpha keratins make up the structural class of proteins, as they reside in the fiber cortex of hair. The second type is the beta keratins, which are a non-extractable, less-studied class of keratins. These are usually present in the hair cuticle and perform protective functions. The third type is the gamma keratins, which have a high sulfur content; these keratins are ~15 kDa in size. Their size is comparatively smaller than the other classes of keratin. These keratins help to maintain the cortical superstructure by cross-linking the disulfide bonds in the hair (Cheng et al., 1995; Gupta et al., 2002; Prakasham et al., 2006). All these types of keratins can be degraded by the enzyme keratinase, which belongs to a class of protease enzymes. Proteases, which account for 60% of the world's marketed enzymes, is responsible for many applications, such as detergents, food, and leather processing (Suntornsuk and Suntornsuk, 2003; Călin et al., 2017; Adetunji and Adejumo, 2018; Kalaikumari et al., 2019).

The enzyme keratinase (E.C. 3.4.99.11) is one of the serine hydrolase groups that disrupt the disulfide hydrogen bonds in the keratin proteins (Cavello et al., 2015; Bohacz and Korniłłowicz-Kowalska, 2019; Kalaikumari et al., 2019). According to UniProt results, one of the protein keratinases produced by *Bacillus subtilis* contains two domains. The first one is 59 amino acids long and encodes for inhibitor I9; the other one is 243 amino acids long and encodes for peptidase S8. The first domain occurs from 19 to 77 amino acid sequences and the second domain occurs from 103 to 345 amino acid sequences. The enzyme also has a metal ion binding site for calcium ion. This means that calcium ions act as cofactors for keratinases; the presence of calcium ions in the media can enhance the activity of keratinases. The structure of keratinase makes it very efficient in its function of degrading keratin proteins (Arora and Mishra, 2016; Moraga et al., 2019). Our daily green waste and animal waste includes plenty of keratins, which remain undegraded due to their complexity. Such insoluble keratins may lead to environmental pollution if left untreated. Thus, as a solution, such wastes are treated by keratinase enzymes, which convert the waste into simpler as well as biodegradable substances (Cavello et al., 2015; Hossain et al., 2017). The extracellular keratinases have been successfully isolated from several microbes by using several fermentation techniques and by optimizing conditions, such as pH, temperature, and type of nitrogen and carbon source and the choice of microbe (Govinden and Puchooa, 2012; Lateef et al., 2015). The keratinases from microbes are effective, biodegradable, and economic and provide much better results as compared to chemical treatments (Manirujjaman et al., 2016; Tamreihao et al., 2017).

## Alkaline Proteases

The genus Bacillus is vital for commercially important alkaline protease (EC.3.4.21-24.99), which is active at alkaline pH ranging between 9 and 11 (Varela et al., 1997; Kocher and Mishra, 2009; Singhal et al., 2012). These alkaline protease producers are distributed in water, soil, and highly alkaline conditions. From a variety of sources, such as detergent contamination (Hsiao et al., 1994; Singh et al., 1999), dried fish (Centeno et al., 1996), sand soil, and slaughterhouses, segregation of alkaline proteases has been stated (Adinarayana et al., 2003). The detergent industry consumes alkaline proteases most abundantly, which are serine proteases with an alkaline pH range (Gupta et al., 2002). These alkaline serine proteases, which are easily inactivated by phenyl methane sulfonyl fluoride (PMSF), account for one-third of the share of the enzyme market (Page and Di Cera, 2008). Alkaline proteases are unique in their activity and maintain a constant alkaline pH while being exploited for different formulations in pharmaceutical, food, and other related industries (Banerjee et al., 1999; Joo et al., 2002, 2004; Dias et al., 2008). A broad range of applications of these alkaline proteases are getting more attention from researchers with the hope of discovering new strains with unique properties and substantial activity (Najafi et al., 2005; Saeki et al., 2007). It is reported that for dehairing of animal skin and hides, *Bacillus* sp. provide the desired hydrolytic, elastolytic, and keratinolytic properties (Bhaskar et al., 2007; Deng et al., 2010; Shankar et al., 2011). These Bacillus strains have been commercially exploited around the globe due to the huge amounts of enzyme secreted with high enzymatic activity (Jacobs, 1995; Ito et al., 1998; Yang J. K. et al., 2000; Beg et al., 2003). Although alkaline proteases are produced by multiple sources (Ellaiah et al., 2002; Prakasham et al., 2005), with the increasing demand of protease in the market, and for cost-effectiveness, only those strains that show greater yield with hyperactivity will be accepted in the current biotechnological advancement (Kumar D. M. et al., 2012). Two essential types of alkaline proteases, such as subtilisin Carlsberg and subtilisin novo are obtained from Bacillus sp., which can be used as an industrial enzyme to produce zein hydrolysates (Miyaji et al., 2006). In halophilic sources, different microbial sp. secreting serine alkaline proteases are also reported (Giménez et al., 2000; Dodia et al., 2008; Vijayaraghavan et al., 2012). The entomopathogenic bacterium *Photorhabdus* sp. strain EK1 (PhPrtPI) containing Ca^2+^ alkaline protease is categorized as a metalloprotease. Owing to its broad-spectrum specificity with different proteins and peptides, it is suggested that PhPrtPI provides nutrients to the nematodes by degradation of insect tissues (Soroor et al., 2009). A *Salinivibrio* sp. strain, AF-2004, produces metallotype protease with a reasonable thermal tolerance and a broad range of pH (5.0–10.0). It is a highly recommended strain due to its thermal and halophilic properties (Amoozegar et al., 2007). Another strain, *Bacillus clausii*, is also recommended for use at a commercial scale for the production of alkaline protease with the use of peptone, Cu, and fructose as the sole source of energy. The optimum pH and temperature recommended is 8–9 and 37–40°C, respectively (Vadlamani and Parcha, 2011). A strain of *Bacillus* sp., MPTK 712, isolated from dairy slush producing alkaline protease exhibits a symbiotic relationship with marine shipworms (Greene et al., 1989; Kumar D. M. et al., 2012). Very rare microbes, such as Kurthia spiroforme are also capable of producing alkaline protease (Amoozegar et al., 2007). Some alkaline serine proteases recognized by goat skin metagenomics library shows homology to peptidases (Vadlamani and Parcha, 2011) and *Cryptococcus aureus* shows good bioactivity with optimum temperature (45–50°C) and pH (9–10) (Kumar D. M. et al., 2012). Different mushrooms producing alkaline protease are also reported (Steele et al., 1992; Li et al., 2009; Pushpam et al., 2011).

## Acidic Protease

Acid proteases are stable and active between pH 3.8 and 5.6 and are frequently used in soy sauce, protein hydrolysate, and digestive aids and in the production of seasoning material. The optimum pH of acidic proteases is 3–4 and the isoelectric point range is between 3 and 4.5 with a molecular weight of 30–45 kDa (Zheng et al., 2011; Ravikumar et al., 2012; Machado et al., 2016). Furthermore, acid proteases are also exploited for use in clearing beer and fruit juice, improving texture of flour paste, and tenderizing the fibril muscle (Zhang et al., 2010). In comparison with alkaline proteases, these extracellular acid proteases are mostly produced by fungal species, such as *Aspergillus niger* (Sielecki et al., 1991), *Aspergillus oryzae* (Yongquan, 2001), *Aspergillus awamori* (Ottesen and Rickert, 1970), *Aspergillus fumigatus* (Shinmyo et al., 1972), and *Aspergillus saitoi* (Sodek and Hofmann, 1970). Most of the fungal extracellular acid proteases are known as *aspergilla opepsins*. Aspartic proteases are acid proteases consisting of 380–420 long chains of amino acid residues constituting the active site for catalytic activity. These acidic proteases are endopeptidases and grouped into three families: pepsin (A1), retropepsin (A2), and enzymes from Para retroviruses (A3) (Somkuti and Babel, 1967). These three families are placed in clan AA. It is found that A1 and A2 are closely related to each other while members of the A3 family show some relatedness to families A1 and A2. An active site cleft of the members of the pepsin family is located between lobes of a bilobal structure (Pushpam et al., 2011). A great specificity of acidic proteases is exhibited against aromatic amino acid residues located on both sides of the peptide bond. These aromatic amino acid residues with peptide bonds are similar to pepsin but less stringent in action. Broadly, acidic proteases are divided into two groups: (i) pepsin-like enzymes and (ii) rennin-like enzymes produced by *Penicillium, Aspergillus, Rhizopus, Endothia*, and *Mucor* (Tomoda and Shimazono, 1964).

## Neutral Proteases

Neutral proteases are defined as, such as they are active at a neutral or weakly acidic or weakly alkaline pH. Mostly neutral proteases belong to the genus Bacillus and with a relatively low thermotolerance ranging from pH 5 to 8 (Table 1). They generate less bitterness in hydrolysis of food proteins due to a medium rate of reaction; therefore, they are considered more valuable in the food industry. Neutrase is incorporated in the brewing industry due to its insensitivity to plant proteinase inhibitors. On the basis of high affinity toward hydrophobic amino acids, neutral proteases are identified and characterized. During production of food hydrolysate, it is slightly advantageous to control the reactivity of neutral proteases due to low thermotolerance. A divalent metal ion is required for the activity of neutral proteases belonging to the metalloprotease type (Barrett, 1995; Woessner et al., 2000; Chavan and Patil, 2007).

**Table 1:** A comparison among different types of proteases.

**Type of protease**	**pH range**	**Use of proteases**	**Classification**	**Sources**	**References**
Alkaline	9–11	Detergent and leather industry	Serine proteases, subtilisin Carlsberg and subtilisin novo	Mostly produced by bacterial species, such as *A. salinivibrio* sp. strain AF-2004, marine shipworms, *Cryptococcus aureus*, mushrooms, *Bacillus* sp.	Miyaji et al., 2006; Dodia et al., 2008; Patil and Chaudhari, 2009; Soroor et al., 2009; Simkhada et al., 2010a; Vadlamani and Parcha, 2011
Acidic	3.8–5.6	Soy sauce, protein hydrolysate, digestive aids and in production of seasoning material, clearing beer and fruit juice, improving texture of flour paste and tendering the fibril muscle	Aspartic proteases, pepsin (A1), retropepsin (A2) and enzymes from Para retroviruses (A3)	Mostly produced by fungal species, such as *A. niger, A. oryzae, A. awamori, A. fumigatus*, and *A. saitoi*.	Sielecki et al., 1991; Steele et al., 1992; Zhang et al., 2010; Pushpam et al., 2011
Neutral	5–8	Food industry, brewing industry	Neutrase, thermolysin	Genus *Bacillus*	Sodek and Hofmann, 1970

Metalloproteases based on specificity in action are grouped into (i) neutral, (ii) alkaline, (iii) Myxobacter I, and (iv) Myxobacter II. A specificity of neutral proteases is shown for hydrophobic acids and inhibited by a chelating agent, such as EDTA (Ethylenediamine tetraacetic acid). Among different types of proteases, metalloproteases are the most diverse. Thermolysin, a well-characterized neutral protease having a single peptide without disulfide bridges, is produced by *B. stearothermophilus*. It has a molecular weight of 34 kDa. Between the 2-folded lobes of a protein, an essential Zn atom and four Ca atoms are embedded, exhibiting thermotolerance. This thermolysin neutral protease is very stable with a half-life of 1 h at 80°C (Fitzgerald et al., 1990; Dawson and Kent, 2000).

## Sources of Proteases

Owing to the high demand of proteases in the global market, the search for proteases has tremendously increased, as they are found everywhere in nature, namely, in plants, animals, and microbes. However, production of plant proteases, such as bromelain, keratinases, and ficin, is time-consuming (Rani et al., 2012). The animal proteases, such as pancreatic, trypsin, pepsin, chymotrypsin, and renin are produced and prepared in pure form in large quantities (Weaver et al., 1977; Boyer and Krebs, 1986). The production of proteases from animal sources is insufficient to fulfill the industrial demand worldwide; therefore, scientists have extended their research of producing protease from bacterial sources (Table 2). Owing to the broad-spectrum biochemical variety and easy genetic manipulation, microbes produce an exceptionally promising number of proteases (Godfrey and West, 1996a; Kuhad et al., 2011). Among different sources, such as plants, animals, and microbes, proteases are generally produced by microbial sources. Among microbes, *Bacillus* sp. are extensively studied for protease production in a large scale, and they are exploited in various industries like leather, detergent, pharmaceuticals, and textile; some fungal species like *Aspergillus* sp. have been studied thoroughly for the production of alkaline protease (Singhal et al., 2012; Singh et al., 2016; Rehman et al., 2017). A list of microbes producing proteases is given below. Halophilic enzymes are getting more attention in biotechnological applications due to their thermal stability and ability to retain activity under high stress from organic solvents except for pyridine, which inhibits protease activity. The enzyme activities remained the same up to 80% even at 50, 55, and 60°C for at least 30 min (Madern et al., 2000; Margesin and Schinner, 2001; Xue et al., 2012).

**Table 2:** Some commercially available microbial proteases.

**Product trade name**	**Microbial source**	**Applications**	**Supplier**	**Activity**	**Characteristics**
Alcalase	*Bacillus licheniformis*	Detergent, silk degumming	Novozymes, Denmark	Activity: ≥0.75 Anson units/ml. Activated at high temperature from 45°C up to 65°C and moderate pH of 7.0–8.5 Stereoselective hydrolysis of amino esters and selective esters; suitable for hydrolysis of proteins; used in transesterification and transpeptidation	Activated at high temperature, moderate pH. Storage temperature: +2°C to +8°C Storage conditions: freezing conditions Toxicity: harmful State: dark brown liquid Density: 1.25 g/ml
Savinase	*Alkalophilic Bacillus* sp.	Detergent, textile	Novozymes, Denmark	Activity: 12 KNPU-S/g Activated at low temperature from 10°C up to 65°C and high pH of 6.5–11, Stereoselective hydrolysis of amino esters and selective esters; suitable for hydrolysis of proteins, hydrolysis of strained amides	Activated at low temperature, high pH Storage temperature: +3°C to +5°C Storage conditions: not freezing conditions Toxicity: Safe as it is not toxic State: granulate Density: 1,300 g/ml
Esperase	*B. lentus*	Detergent, food silk degumming	Novozymes, Denmark	Activity: 8 KNPU-E/g Activated at temperature up to 55°C and high pH of 8.0–12.5, hydrolysis of internal peptide bonds; characterized by excellent performance at elevated temperature and pH	Activated at low temperature, high pH Storage temperature: +0°C to +10°C Storage conditions: not freezing conditions Toxicity: safe as it is not toxic State: liquid Density: 1,070 g/ml
Biofeed proteases	*B. licheniformis*	Feed	Novozymes, Denmark	Activity: ≥2.80 Anson units/ml, pH 9.0, temperature from 15 to 80°C	Acid stable proteases State: liquid, storage at 25°C
Durazym	Protein engineered, variant of Savinase®	Detergent	Novozymes, Denmark	Activity: ≥8.39 Anson units/ml, pH 7–12, temperature from 20 to 80°C while the activity measured at 60°C was regarded as the 100% value	Density 800 g/ml, size of active varies from 18 to 90 kDa, granulates and liquid form, crystalline enzyme
Neutrase	*Bacillus amyloliquefa-ciens*	Upgrade proteins of animal and vegetable origin	Novozymes, Denmark	Activity: ≥0.8 Anson units/g, optimum activity around pH 8 and 20–80°C	Store at 2–8°C State: liquid
Novozyme 3403	*B. licheniformis*	Denture cleaner	Novozymes, Denmark	Type XII-A, saline solution Activity: ≥500 units/mg protein (biuret), optimum activity around pH 8 and temperature 20–90°C	Store at −8°C State: liquid
Novozyme 4551	*B. licheniformis*	Leather	Novozymes, Denmark	Activity: 500 units/mg protein, 93–100% (SDS-PAGE), optimum activity around pH 6.9 and temperature 20–80°C	Store at 2–8°C State: lyophilized powder
Protease	*B. licheniformis*	Food, waste	Solvay Enzymes GmbH, Germany	Activity: ≥2.4 units/g, active between pH 6.5 and 8.5 and has an optimum temperature of 60°C	Store at 2–8°C State: aqueous solution
Proleather	*Alkalophilic Bacillus* sp.	Food	Amano Pharmaceuticals Ltd.,	Activity: ≥3.5 units/g, active between pH 4.5 and 5.5 and has an optimum temperature of 70°C	Store at 2–10°C State: liquid
Protease P	*Aspergillus* sp.	Not specific	Amano Pharmaceuticals Ltd.,	Activity: ≥0.5 units/g, active between pH 4 and 7.5, and has an optimum temperature of <60°C	Store at 3–5°C State: liquid

## Protease and Substrate Specificity

A number of techniques are being exploited for enzyme production from a dominant microbial source for economic improvement (Eichler, 2001; Haki and Rakshit, 2003), but a quest for good quality grade enzymes for industrial use from bacteria is still under consideration. The use microbial origin proteases in the industrial sector is limited by their quality and cost. The increasing interest in using proteases for the production of various eco-friendly goods in the market is of immense importance, and to make the products cost-effective, scientists are in search of a cheap substrate for enzyme production. Almost two-fourths of production cost is due to microbial growth substrate (Singh et al., 2015; Hamza, 2017a). Both solid substrate and submerged fermentation are exploited for the cost-effective production of microbial proteases. The easily available substrate wheat bran is found to be more promising for protease production in solid substrate fermentation (Priya et al., 2016; Hamza, 2017a,b). Other cheap sources of substrate, such as cow dung, agro-industrial waste, groundnuts, and wheat bran can be remarkable for the production of proteases (Krishnaveni et al., 2012; Verma and Agarwa, 2016; Hamza, 2017a). Additionally, other readily available sources of substrate, such as molasses from sugar industry waste, dairy sludge, and effluents are interestingly promising for value-added product enzyme production and concurrently help to lessen eco-pollution (Prabhavathy et al., 2012; Chatterjee et al., 2015; Rao et al., 2017; Corral et al., 2018). For the commercial production of various enzymes especially proteases, waste from the agriculture industry is expected to be used in the future.

## Protease and Yield Improvement

Apart from the use of different substrates for protease production from microbial sources to make them high quality and cost-effective, genetic manipulation provides researchers a new opportunity to make changes in bacterial genome using various biotechnological tools to enhance the yield of proteases with desired characteristics. The diversity in microbes and tools opens a new path for strain improvement for industrial use as well (Rathakrishnan and Nagarajan, 2012; Aruna et al., 2014). Scientists have incorporated different ways to improve protease yield for industrial use, such as cloning and overexpression, screening of strains, fed batch, and chemostat fermentation. Different statistical approaches, such as response surface methodology have also been used for the optimization of different media and growth conditions. Both conventional (UV or chemicals) and modern (rDNA) technology are also used for strain improvement for hyperproduction of proteases (Kumar D. M. et al., 2012; Homaei et al., 2016; Rehman et al., 2017). The rDNA technology is recombinant DNA technology carried out through the combination of our desired gene and the genome of organisms like microbes, plants, and/or animal cells. The new cell (plant, microbes, or animals) produced transgenic organisms called genetically modified organisms. The proteases produced through the transformation of protease genes through microbes like Escherichia coli are called recombinant proteases. Due to thermal instability and the high cost of recovery of enzymes, proteases have been restricted for use in the industry regardless of their advantages. These concerns led to the use of immobilization technology to attempt to increase thermal tolerance, stability to pH, and organic solvents. Immobilization technology has been employed to obtain a high yield of alkaline proteases against a solid support of matrix (Kalisz, 1988; Rao et al., 1998). The proteases are usually immobilized in the alginate–chitosan beads, which exhibit reasonable stability and good activity at 47°C (Mehde et al., 2018; Xu et al., 2018; Özacar et al., 2019; Xing et al., 2019). Genetic engineering with the aim of hyperproduction of enzyme, cost-effectiveness, and quality helps scientists to capture the biotechnology market worldwide. Bioengineered enzymes with greater stability are being generated in the detergent industry, especially using rDNA technology. Under extreme conditions, the expression of gene encoding for proteases through using different vector systems including pHY300PLK, pKL9610, pFX1, and plasmid may be maintained and expressed in *Bacillus stearothermophilus, B. stearothermophilus, E. coli*, and *B. subtilis* (Roja Rani et al., 2012; Kostyleva et al., 2016).

## Purification of Proteases

After production of enzymes, purification of these enzymes is a very complex process. A number of methods are in line for their purification. Several techniques are applied for the recovery of value-added product enzymes. The choice of technique depends on the source of enzyme, whether it is extracellular or intracellular (Mienda and Yahya, 2011). During the production and purification of enzymes, the basic consideration is to produce end products that are cost-effective and of high value using economical techniques. Usually, the precipitation method is used for protein recovery from a crude biological mixture. Different reagents, such as salts and organic solvents are used. The most common practice is the use of ammonium sulfate for the precipitation of proteins in an aqueous solution of acidic, neutral, or alkaline pH, which develops ammonium under alkaline conditions. But the use of ammonium sulfate for detergent enzymes has been a choice because under low temperatures, the solubility of salt limits the positive precipitating quality of sodium sulfate salt while the ammonium sulfate enhanced the solubility of salts (Sumantha et al., 2006; Naidu, 2011; Prabhavathy et al., 2013). The use of ion exchange (CM-Sephadex, DEAE-Sephadex) and gel filtration chromatography is expedient for the production of purified proteases, such as alkaline, acidic, and neutral from different bacterial sources, such as *Bacillus cereus* AT and *Bacillus circulans* (Kanmani et al., 2011; Annapurna et al., 2012). The preferred technique for the recovery of enzyme formed is the use of dialysis membrane. Ultrafiltration is a pressure-driven separation process that is inexpensive and results in little loss of enzyme activity (Rao et al., 1998; Rani et al., 2012). Such promising techniques like gel filtration are used to determine the molecular mass of proteins using a reference standard of mixture of proteins with known molecular weight (Table 3).

**Table 3:** Protease list isolated from various organisms with their molecular weight and classes.

**Proteases**	**Class**	**Occurrence**	**Molecular weights**	**Subunit**	**References**
Tripeptidyl peptidase II	Ser	Cytosol	>1,000,000	Many identical subunits 135 kDa	Tomkinson et al., 1987; Schomburg and Salzmann, 1991; Kloetzel, 2004
Leucine aminopeptidase	Metallo	Cytosol	360,000	Hexamer 6 ×54 kDa or 3 × two subunits, 53 and 65 kDa	Taylor et al., 1984; Kohno et al., 1986; Weston, 2005; Stack et al., 2007
Calpain I/II	Cys	Cytosol	110,000	Heterodimer 80 and 30 kDa	Suzuki, 1987; Melloni and Pontremoli, 1989; Goll et al., 1992; Berchtold et al., 2000
Multicatalytic proteinase	Cys or Ser	Cytosol	700,000	>10 different types of subunits, 22–34 kDa	Rivett, 1989; Driscoll et al., 1993; Hershko and Ciechanover, 1998
Megapain/UCDEN*	Unknown	Cytosol	1,500,000	Many different subunits, 34–110 kDa	Fagan et al., 1987; Hough et al., 1988; Biolo et al., 1995
Endopeptidase 24.11	Metallo	Plasma membrane	90,000	Dimer	Kenny, 1986; Perkins et al., 1999; Keil, 2012
Meprin	Metallo	Plasma membrane	360,000	Tetramer	Bond and Beynon, 1986, 1995
ATP-dependent protease	Ser	Mitochondria	550,000–650,000	Unknown	Desautels and Goldberg, 1982; Watabe and Kimura, 1985; Rock and Goldberg, 1999
Proteinase yscE (multicatalytic proteinase or proteasome)	Cys and Ser	Cytosol	600,000–700,000	Many different types of subunits, 22–33 kDa	Achstetter et al., 1984; Kalisz, 1988; Kleinschmidt et al., 1988
Proteinase ysc Y aminopeptidase	ATP metallo	Cytosol	>600,000–640,000	Unknown	Tanaka et al., 1988; Gomes et al., 2006; Guillaume et al., 2010; Aiken et al., 2011

## Comparison Among Acidic, Neutral, and Alkaline Proteases

It has been studied extensively that among all enzymes, proteases are being used in various industries abundantly, mainly those of bacterial origin. Acid proteases are obtained from fungal species and neutral proteases are of plant origin. Isolation of both acidic and neutral proteases from fungi and plants is labor-intensive and uneconomical comparatively, while alkaline proteases obtained from bacterial species are demanded by industries because of their cost-effectiveness, ease of production, ready susceptibility to genetic manipulation, less labor intensiveness, and limited space for cultivation.

## Microbial Proteases and Industry

Proteases of microbial origin are considered the most significant hydrolytic enzymes, whereas alkaline proteases are ranked the highest in the enzyme market (Mukesh et al., 2012; Mahajan et al., 2016). Interest in studying the proteases has increased not only due to the regulation of different metabolic processes but also due to the significant use in industrial community. The microbes producing substantial numbers of extracellular proteases are of great importance for the industry, and few products of alkaline protease are successfully marketed (Gupta et al., 2002; Gupta and Ramnani, 2006; Vijayaraghavan et al., 2014). Microbial proteases have numerous applications in different industries listed below.

## Protein Hydrolysis

In the food industry, proteases are utilized for modification, palatability, and storage life of all available sources of proteins. High nutritional value preparations of protein hydrolysates are achieved by the use of alkaline proteases. In meat tenderization, alkaline proteases of microbial origin are of immense importance (Rao et al., 1998; Sumantha et al., 2006).

## Food and Feed Industry

**Figure 1:**
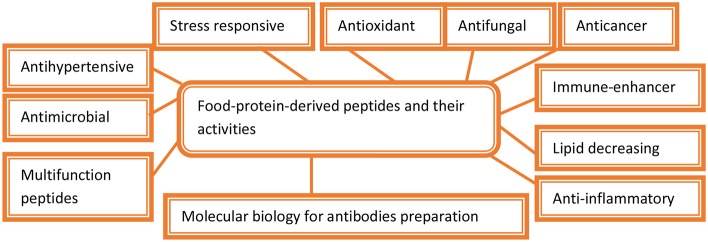
Food-protein-derived peptides and their roles.

During cheese production from milk, proteases are added to hydrolyze kappa casein to prevent coagulation by stabilizing micelle formation. In the baking industry, for quicker preparation of dough, its gluten is partially hydrolyzed by a heat-labile fungal protease because of its early inactivation in subsequent baking. Protein hydrolysate preparation with high nutritional value has been accomplished by the addition of microbial alkaline proteases. The bioactive peptides play an important role in various pharmaceutical drug formations and as potential molecules under stress environmental conditions (Figure 1). This preparation of hydrolysate is vital in infant food formulation and fortification of soft drinks and juices (Ray, 2012; Singhal et al., 2012; Mótyán et al., 2013; Singh et al., 2016). The mackerel hydrolysates helped in the hydrolysis of protein molecules into free amino acids including carosine, anserine, and other small peptides through the use of proteases. The hydrolysis of proteins into amino acids caused the formation of antioxidants that inhibit autoxidation of linoleic acid and the scavenging effects for α,α-diphenyl-β-picrylhydrazyl free radicals (Wu et al., 2003; Li et al., 2008; Gómez-Guillén et al., 2011). It was found that the long peptides with 1,400 Da molecular weight were stronger antioxidants as compared with smaller peptides with molecular weights of 200 to 900 Da (Clemente, 2000; Foegeding et al., 2002; Tavano, 2013). It has been found that the formation of extensive protein hydrolysates through sequential actions of exoproteases and endopeptidases coupled with the release and development of the post-hydrolysis processes was considered as the most efficient way to produce protein hydrolysates that showed well-defined characteristics during protein hydrolysis (Sarmadi and Ismail, 2010; Chalamaiah et al., 2012; He et al., 2013; Power et al., 2013). The bioactive peptide produced from the hydrolysis of various food proteins plays an important role as antioxidants in cell (Thiansilakul et al., 2007; Nalinanon et al., 2011; Kittiphattanabawon et al., 2012). The protein hydrolysates showed excellent solubility, because of which the antioxidant activities of protein hydrosylates were enhanced (Kumar N. S. et al., 2012; Intarasirisawat et al., 2013; Chi et al., 2015). The bioactive peptides show anticalmodulin, anticancer, and hypocholesterolemic properties, and there are also multifunctional properties of the food-protein-derived peptides (Phoenix et al., 2012; Nicolia et al., 2014; Udenigwe, 2014; Nongonierma and FitzGerald, 2015; Agyei et al., 2016).

## Waste Management

The use of chemicals in industries is detrimental to the environment and the surroundings. This hazardous use of chemicals begs for an alternative ecofriendly way for the treatment of waste management. Feathers of poultry containing a very rigid keratin structure accounts for 5% of the body weight and is a rich source of proteins for feed and food. Poultry waste can be degraded into feed and food by the keratinolytic process (Neklyudov et al., 2000; Lasekan et al., 2013). For depilation and cleaning of hairs from drains and clogged pipes, a formulation containing hydrolytic enzymes isolated from B. subtilis, B. amyloliquefaciens, and Streptomyces sp. has been prepared and patented as Genex (Blanch and Moo-Young, 1985; Drew et al., 1985; Ichida et al., 2001; Lasekan et al., 2013).

## Leather Industry

Increased application of alkaline protease at emerging leather industries is due to the elastolytic and keratinolytic activity. These influential properties of alkaline protease are very effective in leather processing industries. The particular uses of protease are found to be relevant in the soaking, bating, and dehairing phase of preparing skin and hides. Extermination of unwanted pigments by enzymatic measures helps in clean hide production. Enzymatic proceedings of pancreatic proteases rely on the bating system. Microbial alkaline proteases have become very popular in leather industries (Takami et al., 1992; Brandelli et al., 2010).

## Detergent Industry

Proteases have been widely used at commercial scale in the detergent industry. The various products in the detergent industry containing proteases as an essential component or ingredient have been used for cleaning of household laundry, dentures, or contact lenses. Of the total sale of enzymes, the utilization of proteases in the detergent industry accounts for ~20%. In 1913, the very first enzymatic preparation, “Brunus,” was prepared consisting of crude pancreatic extract and sodium carbonate. This enzymatic preparation was first marketed in 1956 with a trade name of BIO-40. Alcalase with a trade name of BIOTEX produced by *B. licheniformis* was introduced into the market by Novo industry A/S in 1960 (Jacobson et al., 1985). Protease produced by *B. cereus* BM1 was reported as a good detergent ingredient and shows stable activity in a solution of 10% (w/v) commercial detergent (Fabs Perfect), which suggests its commercial consumption (Varela et al., 1997; Illanes, 2008). Isoelectric point is important for the selection of proteases for detergent preparation. Proteases exhibit remarkable results when pH and PI points of these enzymes are approximately concomitant. There are a few other parameters, such as compatibility with surfactants, bleaches and perfumes (De Virgilio et al., 1993; Bayoudh et al., 2000), good activity, optimum pH, and temperature (Aehle et al., 1993; Bech et al., 1993; Kumar et al., 1998; Gupta et al., 1999) ionic strength, stability and removal potential of stain, which have also been considered for the choice of detergent proteases (Beg et al., 2002; Baş and Boyaci, 2007). Traditionally, detergents work at high temperature but the interest has been increased to search and identify alkaline proteases working in a wide range of temperature (Csuk and Glaenzer, 1991; Breuer et al., 2004). Generally, in the presence of bleaching or oxidizing agent, commercially available proteases are not stable. Recently, rDNA technology has been incorporated to produce bioengineered detergent proteases with greater stability and shelf life. By the use of protein engineering, the replacement of few specific amino acid residues has been studied for bleach and oxidation stability of proteases (Oberoi et al., 2001; Sellami-Kamoun et al., 2008; Haddar et al., 2009b). Proteases have been used not only as laundry detergent but also as dishwashing and cleaning detergents both in institutional and industrial sectors (Estell et al., 1985; Shanlin et al., 1997; Bornscheuer et al., 2012).

## Photographic Industry

Alkaline proteases produced by *B. subtilis, Streptomyces avermectnus*, and *Conidiobolus coronatus* have been successfully reported to recover silver from X-ray films, ensuring that the process is eco-friendlier over the use of chemicals (Godfrey and West, 1996b; Wolff et al., 1996; Yang Y. et al., 2000). Silver recovery by the efficient use of thermally stable mutant alkaline protease produced by *Bacillus* sp. B21-2 has also been reported for its potential (Bettiol and Showell, 2002; Dhawan and Kaur, 2007; Araujo et al., 2008).

## Chemical Industry

Various alkaline proteases producing microorganisms, such as *Bacillus pseudofirus* SVB1, *Aspergillus flavus*, and *Pseudomonas aeruginosa* PseA showed substantial results in peptide synthesis due to stability in organic solvents (Nakiboglu et al., 2001; Ahmed et al., 2008; Shankar et al., 2010). Some alkaline protease producing species of *Bacillus* and *Streptomyces* in the water system are active candidates for peptide and organic synthesis (Masui et al., 2004; Jadhav and Hocheng, 2012; Yadav et al., 2015).

## Silk Degumming

A proteinaceous substance, “sericin or silk gum,” must be removed by the process of degumming from raw silk in an alkaline solution of soap conventionally. Alkaline protease is the best choice to remove sericin while not attacking the fiber. It has been proven that fiber break is not amenable, and silk threads are found to be much stronger than when previous traditional treatments were used (Yadav et al., 2011; da Silva et al., 2017; Radha et al., 2017).

## Medical Field

With the passage of time, scientists have found the broad use of proteases in medical field successfully. In medicine, different formulas, such as gauze, non-woven tissues, and ointment composition containing alkaline proteases produced by *B. subtilis* show promising therapeutic properties (Sen et al., 2011; Anbu, 2013; Awad et al., 2013). Certain lytic enzyme deficiency syndromes are diagnosed to be aided by an oral administration of alkaline proteases (Gupta and Khare, 2007; Joshi and Satyanarayana, 2013). It has been reported that fibrin degradation has been achieved by alkaline fibrinolytic proteases. The use of this fibrinolytic enzyme suggests its future application as an anticancer drug and in thrombolytic therapy (Jaouadi et al., 2011, 2012). Slow-release dosage form preparation containing collagenases with alkaline proteases is extensively used in therapeutic applications. The hydrolysis of collagen by the enzyme liberates low-molecular-weight peptides without any amino acid release for therapeutic use (Romsomsa et al., 2010; Suwannaphan et al., 2017). For the treatment of various diseases, such as burns, carbuncles, furuncles, and wounds, a preparation of elastoterase immobilized on bandage is used (Davidenko, 1999; Palanivel et al., 2013).

## Other Perspectives of Proteases

Apart from vital industrial application of proteases, they are being used for the cleavage of peptide bond to elucidate the association between structure and function of peptides and proteins. Alkaline proteases isolated from *Vibrio metschnikovii* RH530 can be used as an alternative to proteinase K in DNA isolation (Mukherjee and Rai, 2011; Narasimhan et al., 2015; Vijayaraghavan and Vincent, 2015). Hence, the proteases can be viewed as an alternative to many chemicals involved in various biochemical and physiological processes.

## Protease Engineering

Genetic engineering has an enormous contribution on various aspects of life, such as in the field of environmental protection, food production, human health care, animal husbandry, manufacturing of biochemicals, and fuels. In the future, the manipulation of genetic makeup of different organisms will facilitate the production of different therapeutic and industrially important proteins and enzymes to meet the human requirements and combat different serious diseases (Pursel et al., 1989; Cappello et al., 1990; Wang et al., 2003; Mittler and Blumwald, 2010; Hockemeyer et al., 2011).

**Figure 2:**
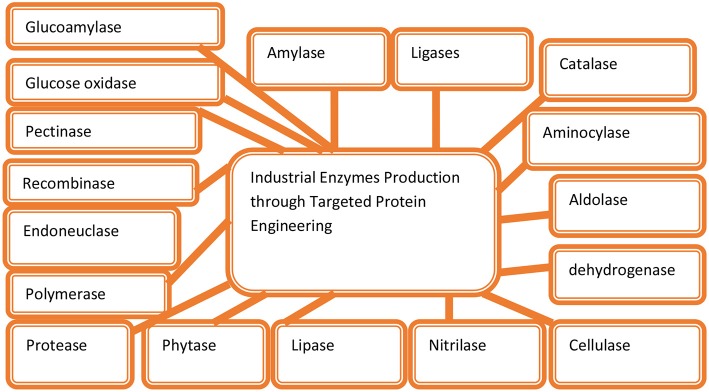
Engineering for various enzymes at the industry level.

The production of genetic modified E. coli for the formation of proteases has introduced new and emerging improvement in the development of recombinant proteins (Figure 2). The use of mutations may also be helpful for the formation and isolation of proteases (Simkhada et al., 2010b; Kotb, 2013). Protease engineering in laundry detergents provided improvement in thermal or high-temperature resistance, which allowed proteases to work even under low-temperature conditions. The three protease engineering campaigns presented provide in-depth analysis of protease properties and have identified principles that can be applied to improve or generate enzyme variants for industrial applications beyond laundry detergents (Barthomeuf et al., 1992; Vijayaraghavan and Vincent, 2015; Vojcic et al., 2015; Coker, 2016; Shahid et al., 2016; dos Santos Aguilar and Sato, 2018). The cold-adapted protease subtilisin has been successfully isolated through evolutionary engineering, which is based on the sequential *in vitro* mutagenesis along with the improved screening method. It was found that the mutation in the subtilisin, termed m-63, exhibited higher efficiency for catalytic activities, which was 100% much higher than that of the wild type at 10°C under N-succinyl-l-Ala-l-Ala-l-Pro-l-Phe-p-nitroanilide as a synthetic substrate for enzyme activities. It was found that the engineering for protease for cold resistance gives cold tolerance in protease, which allowed it to work even under low temperatures (Banerjee and Ray, 2017; Castilla et al., 2017; Onaizi, 2018; Zhou et al., 2019). The mutant proteases from the papain family, such as Glnl9His, Glnl9Glu, and Gin 19Ala, indicated that the Gln19Glu and Glnl9His enzymes participated in the acid-catalyzed hydrolysis in thiomidate, which was converted into amide through the provision of H+ (proton) to form the more reactive protonated thiomidate, which can work at low as well as higher levels of thermal conditions (D'Amico et al., 2002; Siddiqui and Cavicchioli, 2006; Margesin et al., 2007; De Maayer et al., 2014).

Specific inhibition for serin proteases caused crucial switches in a large number of physiological processes for proteases, such as therapeutic applications like ecotin (potential macromolecular inhibitor for serin proteases), which shows as attractive scaffold for engineering the specific proteases inhibitors. The scaffolds showed higher protease inhibition with an apparent dissociation equilibrium constant (Ki^*^) at 11 pM; however, the Ki^*^ values that were related to proteases [Factor Xa (FXa), thrombin, urokinase-type plasminogen activator (uPA), Factor XIa (FXIa), and membrane-type serine proteases 1] showed four to seven higher orders of magnitudes. The adaptabilities of the scaffolds were also demonstrated though isolation for protease inhibitors up to two additional serine proteases, such as Factor XIIa and membrane-type serine proteases 1/matriptase (Liu et al., 2018; Krasileva, 2019a,b; Zhang et al., 2019).

A large number of serin protease subtilisins require the assistance of N-terminal pro-sequence for precursors for the formation of mature and active protease enzymes. The findings from this study indicates that engineering through the use of pro-sequences, i.e., the site-directed or random mutagenesis for proteases, chimeras, and the gene shufflings between the protease members of the serin protease family, would be a very useful tool for the improvement in functions of the autoprocessing protease enzymes. The conventional or traditional protein engineering techniques now have thus far employed mutagenesis in the protease domains for modification in the enzymatic properties of proteases. The new approach, termed pro-sequencing protein engineering, is not only an important technique for the study of protein folding mechanisms but also a highly promising technology to create unique proteases that have various beneficial catalytic properties (Hosse et al., 2006; Ruigrok et al., 2011; Mascini et al., 2012; Fang et al., 2016, 2017a; Verma et al., 2016; Huang et al., 2017). The Gly216 is an active site for proteases and is specific to the MA190 mutant from α-lytic proteases. It has also been found to be extraordinarily tolerant for an amino acid substitution in proteins. The side chains are usually as long as the Trp, which can be accommodated within the substrate binding pocket without decreasing the catalytic activity of enzymes. The GA216 + MA190 expression for specificities of enzymes was altered due to mutation that produced GL216 + MA190 mutants, which were crystallized both with and without a representative in the series of peptide boronic acid transition state that were analog inhibitors for proteases. Results show that the substrates are the agents that specifically determine the α-lytic protease with distributed properties of the active sites and substrate molecules (Cunningham and Agard, 2003; Ljungdahl, 2009; Liu et al., 2014; Fang et al., 2017b; Yang et al., 2017). The proteases are usually not perfect in acyltransferases. All unwanted proteolytic side reactions of proteases and the protease hydrolysis for the acyl-enzyme during kinetic approaches are the key problems for enzymatic peptide synthesis and activity losses. The planning and optimization for enzymatic peptide synthesis always require the “S” or subsite mapping for proteases along with the knowledge of additional fundamental parameters that determined the reaction courses of proteases (Jakubke, 1994; Jäckel and Koksch, 2005; Baker and Numata, 2012; Asgher et al., 2018; Tavano et al., 2018; Antink et al., 2019; de Souza Vandenberghe et al., 2019; Mota et al., 2019; Siar et al., 2019).

Genetic engineering has been instrumental in understanding the relationship between structure and function of different genetic systems and is an excellent method for manipulating the genes. Genetic engineering is being incorporated for the production of industrially important bacterial enzymes. It has been reported that microbial proteases have been isolated and manipulated with the aim of (i) enzyme overproduction, (ii) studying the primary structure of protein, and (iii) applying protein engineering to suit commercial applications. However, the protease gene from bacteria has been cloned and sequenced (Hogdson, 1994).

The ability of *B. subtilis* to be nonpathogenic and to produce extracellular proteins in the medium makes it a potential host for the production of recombinant protease enzyme. *B. subtilis* secretes industrially important proteases subtilisin (apr) or mettaloproteases (npr). This significant study reveals an understanding of the mechanism of overproduction of the proteins. Different strains, such as B. subtilis 168 secretes at least six extracellular proteases into the medium, such as structural genes, neutral protease A and B, minor extracellular protease, bacillopeptidase F, and metalloprotease, which have been cloned. Henner et al. replaced promoters of apr and npr WITH the amylase promoter from *B. amyloliquefaciens* and *B. subtilis*, respectively, to increase the expression (Henner et al., 1985; Sloma et al., 1990; Connelly et al., 2004; Bloor and Cranenburgh, 2006; El-Gamal et al., 2012; Idbeaa and Omar, 2016).

A serine protease gene (hspK) of 90 kDa was cloned and sequenced from B. subtilis (Natto) 16 (Yamagata et al., 1995; Satyanarayana et al., 2012; Guleria et al., 2016). A conserved sequence was found between subtilisin BPN and subtilisin Carlsberg from *B. amyloliquefaciens* and *B. licheniformis* in the coding region and must have a common precursor (Narhi et al., 1991; Li et al., 2013; Souza et al., 2015). It was also reported that the gene encoding subtilisin amylosacchariticus from *B. subtilis* subsp. and sequence showed homology to subtilisin E from *B. subtilis* 168. This gene was then expressed in *B. subtilis* ISW 1214 using a vector pHY300PLK and showed 20 times more activity than the host (Vasantha et al., 1984; Bordusa, 2002; Gamblin et al., 2008; Heck et al., 2013).

Serratia, a gram-negative bacterium, secretes extracellular protease into the medium. Different strains of Serratia like E-15 produce extracellular metalloprotease, which is used as an anti-inflammatory agent. The corresponding gene was expressed in both *S. marcescens* and *E. coli* and an active site and three zinc ligands were revealed. Another study showed that the extracellular serine protease of *S. marcescens* was excreted through the outer membrane of *E. coli*. The nucleotide sequence suggested that it produced a preproenzyme of 112 kDa composed of N-terminal sequence and C-terminal sequence (Stabile et al., 1996; Chalker et al., 2009; Dumas et al., 2013).

In the detergent industry, normally, alkaline proteases are preferred over subtilisin with an optimum pH between 8.5 and 10.0. The ale gene was cloned and sequenced, encoding alkaline elastase YaB based on the information available on enzymes (De Vos, 1987; Rao et al., 1998; Sørvig et al., 2005). The resulting amino acid sequence was 55% similar to subtilisin BPN. The positively charged residues are present on the surface of the alkaline elastase YaB molecule, which facilitates its binding to elastin. Another amino acid sequence of alkaline serine protease deduced from B. alcalophilus PB92 shows homology to YaB. Using chromosola integration, the cloned gene was further used to increase the protease production by gene amplification. An ISP-1 encoding gene isolated from alkalophilic *Bacillus* sp. strain NKS-21 was characterized. It was determined that its nucleotide sequence showed 50% homology to the gene encoding ISP-1 isolated from *B. subtilis, B. polymyxa*, and *Bacillus* sp. strain 221 (Kaneko et al., 1989; Gupta et al., 2008; Deng et al., 2010).

A species of lactobacillus, such as Lactococcus lactis is used as starter culture in the dairy industry, having a complex system of proteolysis that enables it to grow in milk by the degradation of casein into small peptides and free amino acids. This activity leads to the development of flavor and texture of different dairy products. Lactococcal proteases have been classified into P-I-type protease and P-III-type protease on the basis of differences in caseinolytic specificity. The former degrades predominantly beta casein while the latter degrades alpha S1-, beta-, and K-casein (305), but genetic studies focus more on the P-I-type protease. These protease genes located on plasmids greatly differ in size and genetic organization in different strains (Yamagata et al., 1995; Rao et al., 1998; Helianti et al., 2018; Jeong et al., 2018; Ariyaei et al., 2019).

Extracellular serine proteases A and B are secreted by an organism, *Streptomyces griseus*, used for commercial production of pronase. The genes encoding protease A (sprA) and protease B (sprB) were isolated from the *S. griseus* genomic library, and their proteolytic activity was demonstrated in Streptomyces lividans (Henderson et al., 1987; Ramesh et al., 2009; Thirumurugan and Vijayakumar, 2015). Each enzyme is initially secreted as a precursor as suggested by the DNA sequences, which is then incorporated to remove N-terminal propeptide from the mature protease. A strong homology between their coding regions is reported, which suggests that both genes must have originated by gene duplication. Protease B is reported to be one of the major proteases secreted by *S. griseus* ATCC10137, expressed its gene in *S. lividans* (Hwang et al., 1993; Tammawong, 2005).

The extracellular enzyme alpha-lytic protease representing the family of trypsin in a soil bacterium Lysobacter enzymogenes 495 is of particular interest. S1 mapping and nucleotide sequence of the structural gene for alpha-lytic protease from L. enzymogenes 495 suggested that it is synthesized as preproprotein with a size of 41 kDa and is processed to its mature extracellular form (20 kDa) (Vasantha et al., 1984; Silen et al., 1988; Palumbo et al., 2003; Qian et al., 2009). Fusing of the promoter and signal sequences of *E. coli* phoA to the proenzyme portion of the alpha-lytic protease gene was expressed in *E. coli* for protease enzyme production (Silen et al., 1989; Rattenholl et al., 2001; Mitsuiki et al., 2004). With the following induction, an active enzyme was produced both intra- and extracellularly. Fusion of the mature protein domain alone resulted in the production of an inactive enzyme, indicating that the large N-terminal pro-protein region is necessary for activity. Epstein and Wensink also cloned and sequenced the gene for alpha-lytic protease, a 19.8-kDa serine protease secreted by *L. enzymogenes* (Qian et al., 2009, 2014). The nucleotide sequence contains an ORF that codes for the 198-residue mature enzyme and a potential prepropeptide, also of 198 residues (Epstein and Wensink, 1988; Reichenbach, 2006; Wang et al., 2013).

## Future Prospects

The study of biochemical and molecular aspects of proteolytic systems, such as proteases is gaining interest from researchers due to different reasons. Researchers and engineers are looking for robust and novel bacterial enzymes because of the realization of the commercial value of this enzyme. In the future, protein engineering will play a primary role in producing proteases with new properties. Among proteases, alkaline bacterial proteases play a vital role in different industries due to their potential, and their future use is likely to be increased. Advance strategies like protein/genetic engineering, molecular biology, and computational biology are being adopted by the researchers to generate improved protease-producing strains. Bacterial strains with desirable characteristics will be produced by using *in vitro* evolutionary changes in the protein primary structure. One major goal of scientists is to achieve bacterial proteases with characteristics, such as yield improvement, changing substrate specificity, enhancement of thermal stability, altering optimum pH, and prevention of auto-proteolytic inactivation.

## Conclusions

Since the advent of enzymology, enzymes have been broadly utilized in a wide range of industries like textile, pharmaceuticals, leather, food, and detergent. Globally, its use and production are increasing with the use of cheap raw material and by incorporating genetic manipulation. Now, there is an urgent need for the use of such technology that promises cleaner production as an alternative to the use of hazardous chemicals, such as proteases. The higher-ups and the state should take the responsibility of encouraging investors for a cleaner production to mitigate the risk of eco-pollution.

## Author Contributions

AR and SS wrote the initial draft of the manuscript. QA made all necessary corrections and carried out final editing of manuscript. AA, MS, and AM proof read the manuscript. Final approval for publication was given by MA.

### Conflict of Interest Statement

AA was employed by the company Four Brothers Private Limited, Pakistan. The remaining authors declare that the research was conducted in the absence of any commercial or financial relationships that could be construed as a potential conflict of interest.
